# Preclinical murine tumor models: A structural and functional perspective

**DOI:** 10.7554/eLife.50740

**Published:** 2020-01-28

**Authors:** Marion V Guerin, Veronica Finisguerra, Benoit J Van den Eynde, Nadege Bercovici, Alain Trautmann

**Affiliations:** 1 Université de Paris, Institut Cochin, INSERM, U1016, CNRS, UMR8104, F-75014ParisFrance; 2 Ludwig Institute for Cancer Research, de Duve Institute WELBIOUCLouvainBrusselsBelgium; PfizerUnited States; Keio University School of MedicineJapan

**Keywords:** transplanted tumors, spontaneous tumors, TGFβ, vascularization, EMT, microenvironment

## Abstract

The goal of this review is to pinpoint the specific features, including the weaknesses, of various tumor models, and to discuss the reasons why treatments that are efficient in murine tumor models often do not work in clinics. In a detailed comparison of transplanted and spontaneous tumor models, we focus on structure–function relationships in the tumor microenvironment. For instance, the architecture of the vascular tree, which depends on whether tumor cells have gone through epithelial-mesenchymal transition, is determinant for the extension of the spontaneous necrosis, and for the intratumoral localization of the immune infiltrate. Another key point is the model-dependent abundance of TGFβ in the tumor, which controls the variable susceptibility of different tumor models to treatments. Grounded in a historical perspective, this review provides a rationale for checking factors that will be key for the transition between preclinical murine models and clinical applications.

## Introduction

Murine tumor models have proven to be useful for our understanding of biological processes that take place during tumor growth, and for the preclinical development of antitumor therapies. However, the limitations of these models are too frequently overlooked. As a result, numerous anti-tumor drugs that were shown to be efficient in mice ended up being unusable against human tumors. Even if such a major failure has already been discussed, this problem still needs to be examined in depth. A large part of this review will be devoted to comparing the properties of spontaneous (SP) and transplanted (TP) tumors. We will take advantage of two models (a mammary tumor and a melanoma), in which TP and SP tumors can be obtained with isogenic tumor cells. This comparison will be made for the first time by taking into account global structural and functional points of view. Structural features are those that affect the tumor architecture, which is largely dependent on the pre-existence of an epithelial to mesenchymal transition (EMT). For the functional analysis in TP and SP tumors, we examine a series of key tumor features: vascularization, growth rate, immune infiltrate and inflammation, tumor metabolism, and influence of tumor-derived TGFβ. We evaluate the influence of these features on spontaneous growth rate and, most importantly, on the sensitivity of tumors to various treatments, as summarized in Table 1.

**Table 1.** Structural and functional comparison of SP and TP tumors.

		SP vs TP	Subcutaneous (s.c.) TP vs orthotopic (ortho.)TP	References
Vasculature fragility		TP > SP	Ortho > s.c.	Fenton et al. (2001); Drees et al. (2015); Guerin et al. (2019); Fung et al. (2015)
Vasculature growth rate		TP > SP		This review
Tumor architecture		SP > TP SP = TP		This review Weiss et al. (2017); Guerin et al. (2019)
Tumor growth rate		TP > SP TP = SP		Wexler et al. (1965); McCredie et al. (1971); Rous (1914); Burton and Begg (1961); Zhu et al. (2017)
TGFβ abundance		SP > TP	Ortho > s.c.	Guerin et al. (2019); Zhu et al. (2017); Choudhury et al. (2004)
Spontaneous immune response		TP > SP		Anders et al. (2017)
Tumor-associated macrophage (TAM) renewal rate		TP > SP		Strachan et al. (2013); Franklin et al. (2014)
Myeloid-derived suppressor cell (MDSC) abundance		SP > TP	Ortho > s.c.	Coffelt and de Visser (2015); Zhu et al. (2017); Guerin et al. (2019); Devaud et al. (2014)
Sensitivity to	Immunogenic cell death (ICD)-based chemotherapy	TP only		Ciampricotti et al. (2012); Coffelt and de Visser (2015)
	Stimulator of interferon genes protein (STING) agonists	TP only		Guerin et al. (2019)
	Adoptive transfer of T cells	TP > SP		Zhu et al. (2017)
	Vaccination	TP only		Old et al. (1961); Hirsch (1962); Reilly et al. (2000); Ercolini et al. (2003)
	Cortisone	TP > SP		Burton and Begg (1961)
	Interleukin-12 (IL-12)	TP only		Lee et al. (2002)

The fact that different tumor models may not be equivalent is an issue that was first recognized a long time ago. One of the first to address this problem was Francis Peyton Rous, well before the Nobel prize that he obtained in 1966 for his work on virus-induced tumors. In 1914, FP Rous examined the extent to which the mouse diet could influence tumor growth rate in SP and TP tumors (Rous, 1914). He concluded that starvation slows down tumor growth, by affecting both the tumor and its stroma. Importantly, he pointed out that results obtained in these SP and TP systems differ and that 'generalizations from work with transplanted tumors as regards the effects of diet on spontaneous growths are unwarranted’.

Since then, the specific features of different tumor models have been discussed in several reviews (Sharpless and Depinho, 2006; Talmadge et al., 2007; Coffelt and de Visser, 2015; Olson et al., 2018), often with a focused scope, such as a comparison between the different types of genetically engineered mice models (GEMM) designed to develop so-called ‘spontaneous’ tumors. Before starting the comparison between TP and SP tumors, it is necessary to have a global vision of the wide variety of murine tumor models.

## Variety of transplanted tumors

The diversity of TP tumors stems from the *origin* and the *type of cells* that are implanted, their *abundance* and their *site of implantation* (subcutaneous or orthotopic). These tumor cells may be established cell lines, primary tumor cells freshly dissociated from spontaneous tumors, or fragments of tumors transferred from donor to host animals. In the latter two cases, transplanted cells include different cell types present in the tumor microenvironment. In the two isogenic models that are examined in this review, the TP tumors developed after implantation of a tumor cell line in one case (the melanoma), whereas TP tumors in the PyMT mammary model developed after transplantation of dissociated SP tumors.

### Syngeneic tumors versus xenografts

In a vast majority of studies with TP tumors, tumor cells are grafted in syngeneic mice. If a treatment proves to be efficient in such preclinical settings, the translation to clinical studies often presents difficulties, because the molecular tools developed in the murine models cannot always be used for humans. For instance, the STING agonist DMXAA (5,6-dimethylxanthenone-4-acetic acid), which destroys tumor vessels in TP murine tumors quite efficiently, is totally devoid of effects against the vasculature of human tumors. This difference is mainly due to the fact that DMXAA action in TP tumors requires STING binding and activation; however, this molecule does not bind at all to human STING (Conlon et al., 2013). This explains the total absence of effects of DMXAA in a Phase III clinical trial involving almost 1300 patients (Lara et al., 2011). Even when compatible with human molecules, STING agonists may still not work in clinical trials given their inefficacy in SP tumors compared to TP models, as will be discussed below. The species specificity of molecular tools is also true for a large panel of antibodies and engineered constructs, such as chimeric antigen receptors (CAR).

For these reasons, pharmaceutical companies have encouraged the use of human xenografts, that is of human tumor cells grafted in immunocompromised mice. Their obvious advantage is the immediate availability for clinical studies of the molecular tools developed in the preclinical studies. The reasoning was sound when the field was dominated by oncologic approaches targeting first tumor cells. However, the obligation to use immunocompromised mice (either RAG^–/–^, *Scid*, NOD or NSG) that will tolerate the xenograft is now considered to be a major drawback. Indeed, most anti-tumoral treatments, including chemotherapy, radiotherapy, or adoptive transfer of CAR T cells, clearly need to stimulate endogenous immune cells for optimal efficacy. In immunodeficient mice, the absence of T cells and, eventually, of additional types of immune cells, severely limits the usefulness of models based on xenografted tumors. This may explain why preclinical models based on human xenografts have failed to predict the clinical efficacy of most of the therapies targeted to cancer-driving proteins (Day et al., 2015).

It is theoretically possible to circumvent this problem by using humanized mice. However, because immune responses involve many different cell types, obtaining a mouse with a fully humanized immune system is very complicated (Olson et al., 2018). Moreover, for humanization to be complete, it should be extended to other cells of the tumor microenvironment, that is endothelial cells and stromal cells. The complexity and the resulting cost of such mice are both huge.

### Subcutaneous versus orthotopic implantation

The advantage of implanting tumor cells subcutaneously is that the size of these tumors is easier to measure than that of visceral tumors. However, numerous papers have shown that tumor growth, vascularization and sensitivity to anti-tumoral treatments are dependent upon the site of implantation. Thus, there are fewer functional blood vessels in orthotopic tumors than in subcutaneous tumors, as measured with a perfusion marker (Fung et al., 2015). Compared to subcutaneous tumors, orthotopic tumors are more immunosuppressive in nature, with a larger abundance of alternatively activated macrophages, and they are less sensitive to immunotherapy than subcutaneous tumors (Devaud et al., 2014). This difference could be related to the fact that TGFβ is present in tumors derived from orthotopic but not subcutaneous implantation, as shown in a pancreatic cell line (Choudhury et al., 2004). Note that TGFβ is frequently present in SP tumors, as discussed by Guerin et al. (2019), and from this point of view, orthotopic TP tumors are more similar than subcutaneous TP ones to SP models.

## Variety of spontaneous tumors

Even if this review is devoted to the analysis of murine tumor models, it is worth remembering that other tumor models could also deserve consideration (Box 1). SP tumors may develop with aging in some strains of non-transgenic laboratory animals that have been selected for being cancer-prone. They constitute a first type of SP or autochthonous tumors. This is the case, for instance, for one-year- old C3H female mice, which often develop one or two mammary tumors (Waxler et al., 1953).

Amongst different strains of laboratory mice, a negative correlation has been established between the probability of developing such tumors and the efficacy of the immune system. This efficacy was evaluated by the strength of the immune responses triggered by sheep red blood cells. This correlation could indicate that cancer formation is a relatively frequent process that is counterbalanced by immune surveillance (Frese and Tuveson, 2007).

Oncogene-induced tumors, which develop in GEMM, constitute a second type of murine SP tumors. A number of reviews have been devoted to the properties of different GEMM, the most frequently used model of SP tumors, for cancer studies ( Frese and Tuveson, 2007; Willimsky and Blankenstein, 2007; Day et al., 2015; Kersten et al., 2017; Olson et al., 2018). Oncogene-induced tumors present a high incidence, which leads to a delay of only a few weeks between birth and tumor development. Different GEMM have been constructed to generate tumors in various organs. For instance, mammary tumors develop ‘spontaneously’ in models where an oncogene (either PyMT or *neu*) is targeted to the mammary glands by the inclusion in the oncogene promoter of MMTV, that is Mouse mammary tumor virus (MMTV-PyMT or MMTV-neu mice).

In MMTV-PyMT mice, up to 10 tumors develop with a delay of 2 months in FVB mice, and of 5 months in C57BL/6J mice. Similarly, oncogene-driven tumors are generated in the prostate in the TRAMP model (Foster et al., 1997) and in the pancreas in the Rip-Tag model (Ye et al., 1994), whereas melanoma tumors are developed in the Ret model (Takahashi et al., 1992). In some variants of these GEMM, such as the TiRP (Tamoxifen-Induced, expressing active Ras and the P1A tumor-specific antigen) melanoma model, the expression of the oncogenes is induced, for instance by tamoxifen (Huijbers et al., 2006; Kersten et al., 2017; Zhu et al., 2017). In practice, inducible models are somehow intermediate between TP and SP oncogene-induced tumors. Like TP tumors, their triggering is sudden, but, as for oncogene-induced tumors that develop with aging, there is no massive introduction of tumor cells, many of which die and trigger an inflammatory situation.

Box 1. Tumor models in different animals.

When considering other animal tumor models, the Sprague-Dawley rat is a strain well-known to develop spontaneous endocrine tumors (Suzuki et al., 1979). There is also a strain of small pigs that have been selected for their propensity to develop spontaneous melanoma, which often regress spontaneously (Oxenhandler et al., 1982; Pérez et al., 2002). The mechanisms underlying this spontaneous regression have never been studied in detail, which is a pity given the proximity of the immune systems of humans and pigs. By contrast, there are animals that almost never develop cancers, such as mole rats, which is remarkable, given their exceptional longevity (frequently >30 years) (Deweerdt, 2014). The analysis of these other animal models is of major interest, but beyond the scope of this review.

One may consider that the incidence of these GEMM tumors is excessively high, and results in multifocal or multiple tumors, which develop much faster than those in humans. This is why T. Blankenstein and colleagues have developed a third model, the so-called sporadic tumor model, which is still oncogene-induced but with such a low probability that only one or two tumors develop, after a long delay in the order of one year. This model is certainly as close as possible to human tumors (Willimsky and Blankenstein, 2007; Willimsky and Blankenstein, 2005).

Given their dependency on specific genetic events, GEMM have proved to be useful for testing the efficacy of agents targeting farnesyl transferase, epidermal growth factor receptor, angiogenesis, matrix metalloproteases and vascular endothelial growth factor (VEGF) to inhibit tumor growth (Van Dyke and Jacks, 2002; Suggitt and Bibby, 2005; Kohl et al., 1995; Lenferink et al., 2000; Bergers et al., 1999; Bergers et al., 2000; Huss et al., 2003). Moreover, GEMM also make it possible to evaluate how mutations in specific genes affect tumor sensitivity or resistance to chemotherapeutic drugs, such as cyclophosphamide in lymphoid malignancy (Schmitt et al., 1999) or doxorubicin and paclitaxel in mammary and salivary tumors (Bearss et al., 2000).

However, by construction, the mutational burden of tumors in GEMM is usually low compared to that observed in human tumors. To reduce this gap, it has been proposed that the mutation load in GEMM should be increased, for instance by introducing mutations in the DNA mismatch repair machinery (Germano et al., 2017). An additional difference between GEMM and human tumors should be noted: a human tumor is expected to correspond to the descendance of one or a few initially transformed cells, whereas in a GEMM, the constitutive expression of a few oncogenes maintains the continuous appearance of newly transformed cells. A single stream is not equivalent to multiple streams that are constantly renewed.

## Carcinogen-induced tumors and metastases

In addition to the TP and SP models, tumors may also be induced by a carcinogen, either chemical or mineral/mechanical (asbestos). In carcinogen-induced models, the type and route of administration of the carcinogen dictates the location in which the tumor is formed. For instance, topical application of DMBA/TPA (7,12-dimethylbenz[a]anthracene/12-O-tetradecanoylphorbol-13-acetate) results in skin carcinogenesis, and injection of methylcholanthrene (MCA) intramuscularly gives rise to fibrosarcomas. Carcinogen-induced tumors show a strong and long-lasting inflammation (Shi et al., 2017) that resembles the chronic inflammatory milieu of GEMM such as the K14-HPV16 transgenic model of skin carcinogenesis (de Visser et al., 2005).

Given the fact that most carcinogens are also mutagenic agents, carcinogen-induced tumors have a higher mutational burden compared to GEMM or TP models, which are most often genetically defined by two or three mutations (Olson et al., 2018). Consistent with the fact that a high mutation load increases the occurrence of neoantigens recognizable by a large number of T cells (Chan et al., 2019), thus influencing the importance of the T cell infiltrate, tumor patients with high mutational burden usually show a good response to immunotherapy (Lawrence et al., 2013; Alexandrov et al., 2013; Quiroga et al., 2016; Chalmers et al., 2017; Schumacher and Schreiber, 2015; Gros et al., 2016; Sharma and Allison, 2015; McGranahan et al., 2016). The higher immunogenicity of carcinogen-induced tumors compared to GEMM tumors has been illustrated by studies showing that sarcomas triggered by KrasG12D oncogene activation and Trp53 deletion are not recognized by the immune system, contrary to the MCA-induced mouse model of sarcoma, which may induce the appearance of tumor-specific T cells (DuPage et al., 2012; Matsushita et al., 2012). In addition, MCA-induced mouse sarcomas harbor genetic mutations similar to those found in patients bearing tumors that are likely to be carcinogen-induced (Liu et al., 2014).

Given the importance of tumor immunogenicity in predicting the response to immunotherapies, carcinogen-induced tumor models would be of interest for investigating the efficacy of immunotherapeutic agents. In addition, and contrary to what happens in TP models, carcinogen-induced tumors and SP tumors share the relative slowness of their development, which allows the formation of a complex tumor microenvironment (Olson et al., 2018). However, carcinogen-induced models have been employed in only a minority of immunotherapy pre-clinical studies. See for instance, a study on the synergistic effect of anti-CD73 and anti-PD-1 treatment in delaying progression of MCA-induced fibrosarcomas (Allard et al., 2013). The delayed development, heterogeneous appearance, location, size and number of carcinogen-induced tumors contribute not only to the biological interest but also to the experimental complexity of these tumor models (Liu et al., 2015). These features of carcinogen-induced models, which are less marked but comparable to those observed with sporadic tumor models, make it difficult to produce homogeneous groups of animals that can be used to compare therapeutic modalities.

Transplanted cell lines that are derived from carcinogen-induced tumors have been more commonly used (Efremova et al., 2018; Redmond et al., 2014; Noguchi et al., 2017). One may expect that such tumor cells have a higher mutational burden than other TP cell lines, and could potentially be more immunogenic. However, the rapid growth of such tumors is typical of TP tumors, with a quickly built microenvironment. This will put limits on their clinical relevance, as further discussed later.

All of the examples given above concern primary tumors, and not metastases, which constitute a problem of major importance because most cancer patients die of metastases, not of their primary tumors. The use of murine models for studying the formation and treatment of metastases is too important and specific a topic to fit within the scope of the present review, and will not be further examined here.

## Comparing TP and SP tumors

Now that the global landscape of tumor models has been recalled, we can begin to compare them. The discussion will focus on comparing TP and SP tumors, by examining the different *functional* aspects of the tumors that appear to be model-dependent. Up to now, such comparisons have mainly dealt with differences in the vasculature of TP and SP tumors (Falk, 1982; Fenton et al., 2001; Fenton et al., 2004; Sikder et al., 2003; Lee et al., 2002). Here, we underline the importance of not only the vasculature but also the tumor's growth rate, immune infiltrate, inflammation level, metabolism, and TGFβ signaling, and how these characteristics influence tumor sensitivity to anti-cancer treatments (as summarized in Table 1). We also highlight the variability observed for different features. This variability does not result from some inaccuracy in the measurements. It rather reflects the importance of stochasticity in life, which has been taken into account in several complementary concepts: first in Darwin’s theory of natural selection of random variants, then in the notions of ‘epigenetic landscape’ (Waddington, 1957), ‘gene network dynamics’, multi-stability and self-organization, which allow a given living system to adopt several possible stable states (Huang, 2012). A phenotype is not determined by genotype alone but also by constraints including stochastic events and epigenetics. Therefore, the ‘variability’ that is inherent to some tumor models should not be concealed.

### Vascularization

Using various approaches (Box 2), several steps in tumor angiogenesis have been distinguished. This process starts with proliferative endothelial cells that sprout from pre-existing blood vessels, giving rise to uncovered blood vessels of small diameter. The newly formed blood vessels subsequently expand by means of endothelial cell proliferation and migration to form larger blood vessels, which are progressively covered by pericytes. Contrary to the initial uncovered sprouts, these larger mature vessels are formed by quiescent endothelial cells that have limited proliferation and sprouting (Gee et al., 2003). However, all of these processes are often deregulated at cellular and molecular levels in cancer, giving rise to a heterogeneous, disorganized and misshaped vessel network, which is aberrantly covered by pericytes and therefore poorly functional (Leite de Oliveira et al., 2011). From tumor to tumor, and for reasons mentioned above, a huge variability is observed in the detailed shape of the vascular network and in the size of its meshes.

Angiogenesis of small tumors is more active than that of large tumors, and the vascular tree is more immature in small tumors. This could explain why small tumors are more sensitive to anti-angiogenic treatments than large ones (Gee et al., 2003). Both SP and TP tumors may be responsive to anti-angiogenic treatments (Fenton et al., 2001), but the response in SP models varies from tumor to tumor (Fenton et al., 2004; Fenton et al., 2005; Guerin et al., 2019). Endoplasmic reticulum (ER) stress is a major consequence of defective tumoral vascularization and resulting hypoxia. This stress, which has been measured in vivo with a bioluminescent probe in a model of multiple mammary primary tumors, displays a marked variability between the different tumors of the same mouse and correlates positively with tumor growth rate (Spiotto et al., 2010).

A fundamental difference between the vascularization of SP and TP tumors is the ‘*angiogenic switch*’ (Folkman and Hanahan, 1991), that is the point at which angiogenesis becomes actively sustained by the tumor microenvironment through the secretion of pro-angiogenic molecules by tumor cells and macrophages, thus perturbing the local balance between anti-angiogenic and pro-angiogenic factors (Folkman, 1971; Folkman, 2002). SP tumors can only grow after this switch has taken place (Hanahan and Folkman, 1996). In TP models, tumor cells have already switched. Therefore, immediately after their implantation, they start to produce the pro-angiogenic factors necessary for blood supply and tumor growth. As soon as 18 hr after implantation, the beginnings of vascularization in TP tumors may be directly observed through a window chamber (Wood, 1958).

Box 2. Analyzing tumor vascularization.

Tumor vessel morphology and functionality can be studied in different ways, for instance by the immunostaining of tumor sections for endothelial cell markers (CD31^+^, Tie-2^+^), alone or combined with markers for proliferation (PCNA^+^) or apoptosis (active caspase 3) that are used to identify proliferating or apoptotic endothelial cells, respectively. Tumor vessel coverage and stability can be investigated by combining endothelial cell staining with laminin or pericyte (α-SMA^+^) staining. Vessel perfusion or vessel leakage can be assessed by intravenous injection of fluorescently labelled lectin or 70-KDa dextran molecules, a few minutes before sacrificing the mice. The perfusion of surrounding tissues can be evaluated by injecting Hoechst 33342 5 min before the sacrifice. The hypoxic probe pimonidazole or the hypoxic marker (CAIX) allows the identification of hypoxic tumor areas. Vessel ultrastructure can be studied by scanning and transmission electron microscopy.

Another difference in the vascularization of SP and TP tumors is related to the multifocal origin of TP tumors after the simultaneous implantation of tens or hundreds of thousands of tumor cells. This multifocality influences the architecture of the vascular network (Falk, 1982).

Compared to slow SP tumors, the rapidly growing TP tumors are likely to have more immature blood vessels that are more sensitive to inflammatory cytokines such as TNFα, which may be produced following a bacterial infection. By inducing vascular disruption, TNFα and other pro-inflammatory cytokines could facilitate bacterial tumor-colonization. The different vasculature structure in TP and SP tumors may explain why, after systemic administration of *Salmonella typhimurium*, which are bacteria with reported tumor-targeting propensity, bacterial colonization was higher in TP than in SP tumors. As a result, *S. typhimurium* may exert an oncolytic action against TP but not against SP tumors (Drees et al., 2015). In line with the observation in SP models, clinical studies showed lack of tumor colonization by the *Salmonella typhimurium* strain VNP20009, again underlying how transplanted tumors do not always mimic the autochthonous tumors that develop in SP models or in patients (Drees et al., 2015).

### Tumor architecture

**Figure 1.**
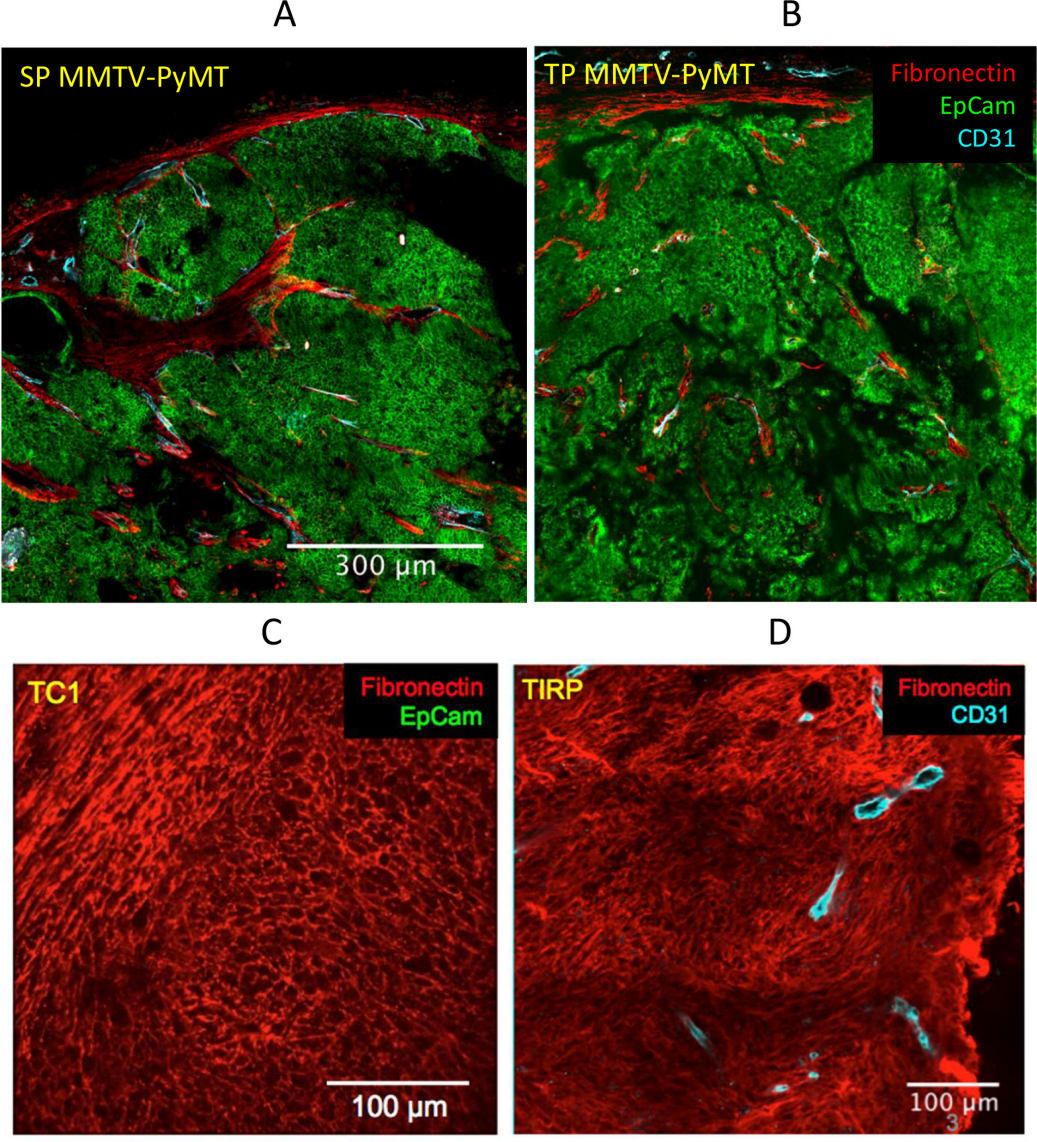
Fibronectin expression is restricted to stromal cells in PyMT tumors, but not in tumors that have undergone EMT. In the MMTV-PyMT model, fibronectin is only expressed by stromal cells, if the tumor is (**A**) spontaneous (SP) or (**B**) results from an orthotopical implant (TP) of cells dissociated from a SP tumor. Fibronectin may also be expressed by all tumor cells after EMT has taken place in (**C**) the TC1 cell line, here transplanted subcutaneously, or in (**D**) a spontaneous melanoma, TiRP. Tumor pieces were fixed overnight with periodate-lysine-paraformaldehyde at 4°C. Then, tumor samples were embedded in 5% low-gelling temperature agarose prepared in phosphate-buffered saline (PBS). 350 μm slices were cut with a vibratome in a bath of ice-cold PBS. Immunostaining of surface markers was performed at 37°C for 15 min with antibodies specific to EpCAM-BV421, CD31-Biot (both BD Pharmingen) or Fibronectin (Abcam). CD31-Biot was revealed with a streptavidin-PE (BD Pharmingen) and fibronectin with a goat anti-rabbit-FITC (Invitrogen).

After tumor initiation, stromal cells may be recruited by the nascent tumor from neighboring normal tissue or may differentiate from mesenchymal cells arriving through the blood from the bone marrow (Bucala et al., 1994; Kraman et al., 2010). The self-organization of the stroma involves different cell types including stromal cells, endothelial cells and macrophages. The stromal compartment usually includes a peritumoral capsule and stromal sheets between tumor islets. This organization is typical of human carcinoma and it is also observed in some murine tumor models (see below). In the MMTV-PyMT (pre-EMT) model, fibronectin is only expressed by stromal cells, and the vasculature is strictly associated with the stroma. This holds true whether the tumor is SP, or whether it results from an orthotopical implant (TP) of cells freshly dissociated from a SP tumor (Figure 1A&B) and thus includes multiple cell types that have not been kept in culture. However, in most commonly used models of TP tumors, 10^5^–10^6^ cells from an established tumor cell line are injected subcutaneously. Cells kept in culture for a long time usually drift, and such tumor cell lines have frequently acquired fibroblast markers such as gp38 or fibronectin because they have gone through EMT. An example is provided by the TC1 (TP) tumor model (Figure 1C). Post-EMT tumor cells never form tumors that are organized into tumor islets and stromal sheets, as if the self-organization of the stromal compartment were hampered by the similarities between fibroblasts and post-EMT tumor cells. Moreover, a mesenchymal transition may sometimes take place in vivo. This is the case for a SP melanoma model named TiRP described in Zhu et al. (2017) (Figure 1D). This tumor shows a uniform fibronectin expression and no tumor islets nor stromal sheets. The same structure is observed in the corresponding TP melanoma (not shown).

Thus, the existence or not of a mesenchymal transition determines a certain architecture of the tumor. Importantly, this structural organization influences the distribution of monocytes/macrophages and T cells. Indeed, this immune infiltrate is concentrated in the stromal sheets when they exist, whereas it is more uniformly dispersed in the tumor mass after EMT (Thoreau et al., 2015; Weiss et al., 2017).

The use of tumor models that present an architecture of tumor islets separated by stromal sheets is of particular relevance for the following reason. M. Angelo and colleagues have recently shown that, in breast cancer, an enriched co-occurrence of immune populations defines a structured immune environment that is indicative of a good prognosis (Keren et al., 2018). They have established an important correlation between a structure (the spatial distribution of immune cells in the tumor) and a function (the efficacy of the anti-tumoral immune response), without suggesting a causal explanation for this correlation. On the basis of the previous discussion, we propose that a well-developed, regular stroma surrounding a functional vascular tree provides an oxygenated space in which immune cells can be recruited. The different cell types gathered there may interact and are ready to respond to an external stimulation. Murine models show that such structures are not observed when tumor cells have gone through EMT. If such a structure is important in human primary carcinoma, one should be aware of the limits of murine models that do not present such an architecture.

### Growth rate

As already pointed out, the growth of a tumor is entirely dependent on its vascularization. Indeed, in the absence of angiogenesis, a tumor cannot grow beyond 1–3 mm in diameter (Greene, 1941; Gimbrone et al., 1969). Once vascularized, the tumor cells that proliferate most actively are those located close to blood vessels (Tannock, 1968). The growth rate of tumor cells is thus indirectly controlled by that of endothelial cells within the tumor (Tannock, 1970).

**Figure 2.**
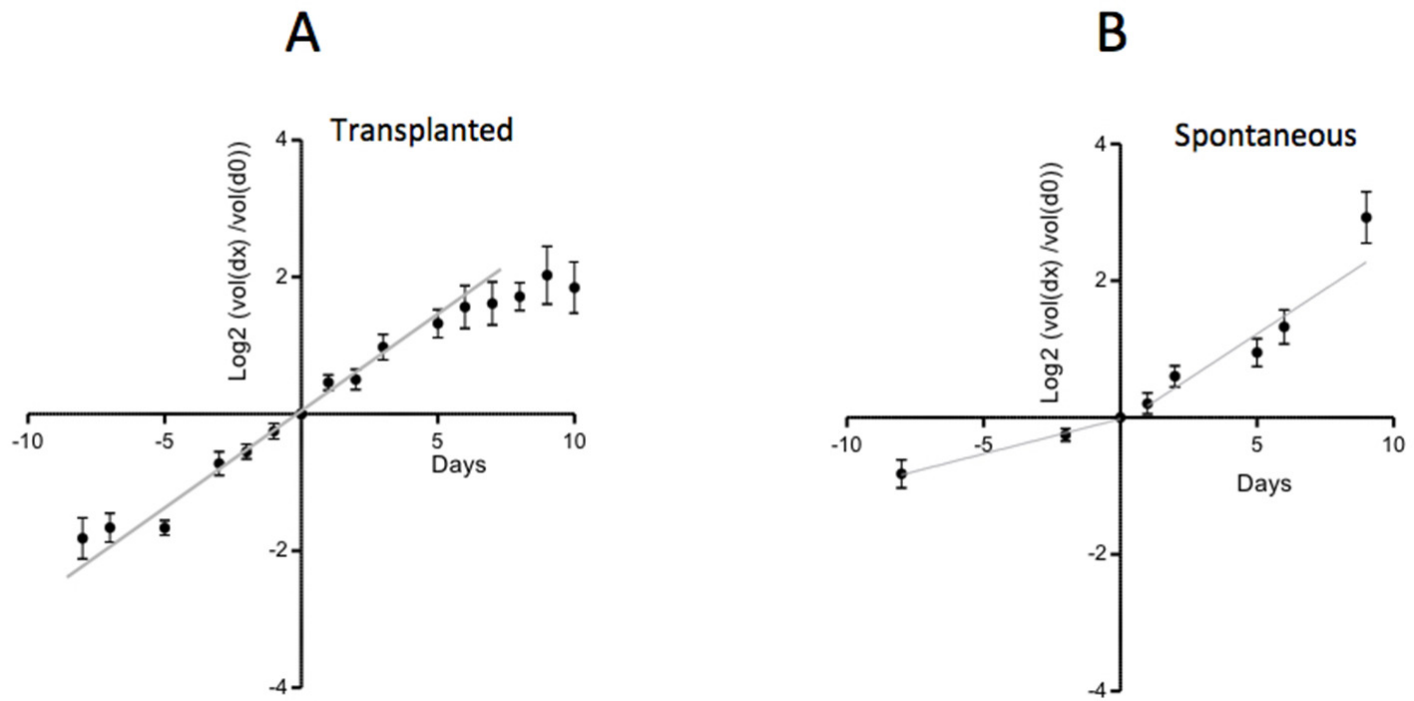
Growth rate of TP and SP PyMT tumors. (**A**) The growth of TP tumors slows down with time. (**B**) The growth of SP tumors, which is initially slow, accelerates with time. Data from 45 tumors from 45 TP mice and 26 tumors from 9 SP mice. The small and large tumor diameters, d and D, were measured with calipers. Tumor volume was approximated by the volume of a spheroid (d^2^.D/2). For each tumor, the volume at time day x is expressed relative to its volume at day 0, when the tumor volume was ~ 50–150 mm^3^. The ratios (volume(dx)/volume(d0)) expressed on a Log2 scale, are represented as a function of time.

Tumor growth rate *diminishes* when tumors outgrow their blood supply (Cataland et al., 1962). Small TP tumors are better vascularized than large ones, in terms of average distance between blood vessels and tumor cells. Indeed, it has been shown that oxygen diffuses with a space constant of a few tens of microns. As a result, tumor cells that are located further than 60–80 µm from a blood vessel suffer from excessive hypoxia, which triggers their apoptosis followed by secondary necrosis (Thomlinson and Gray, 1955; Carmona-Fontaine et al., 2017) (and our own unpublished observations). This partially explains why the growth of TP tumors slows down when tumors are > 1 g (or 1 cm^3^) (Cataland et al., 1962) and Figure 2A. There are examples in which the growth rate is higher for TP than for SP tumors (Wexler et al., 1965; McCredie et al., 1971), but, given the limited number of available comparisons and inconsistent evidence this certainly cannot be considered to be a general rule (Rous, 1914; Burton and Begg, 1961).

Surprisingly, it is also possible, under some conditions, to observe an *acceleration of tumor growth*. Thus, we have observed that SP mammalian tumors have an initial slow growth rate, which increases about two-fold when tumors reach a size of around 6 mm in diameter (Figure 2B), reaching a growth rate comparable to that of TP tumors. The reason for this frequent acceleration of SP tumor growth is unclear. One possibility is that tumor cell proliferation is initially slowed down by different microenvironmental brakes, including TGFβ, which is abundant in these tumors (Guerin et al., 2019). At some point, tumor cells may become unresponsive to TGFβ, as shown in different tumor models (Guerin et al., 2019).

Other examples of accelerations of tumor growth have been reported. For instance, when the size of SP tumors is reduced by surgery, the remaining part of the tumor regrows very rapidly, faster than before surgery (Havas and Donnelly, 1961). This observation is also common for human tumors: a partial tumor regression in response to a treatment is followed by a swift regrowth.

Thus, variations in the rate of tumor growth may be observed in two directions: accelerations of tumor growth for some SP tumors, either spontaneously or after treatment, or a slowdown that may be attributed to an insufficient vasculature, and ensuing hypoxia and necrosis. With exactly the same tumor cells, we have observed either an acceleration in a SP context (Figure 2B) or a slowing down in a TP context (Figure 2A). Such rate changes in tumor growth, combined with the inherent stochasticity involved in tumor formation mentioned earlier, cause important variability in tumor growth rates in murine models.

Altogether, tumor growth rate is strongly dependent on the angiogenesis rate. Tumors that grow fast are not only more aggressive but also more susceptible to antitumoral treatment for several reasons. First their vascular network is more immature and therefore more fragile. Second, rapidly proliferating cells are more susceptible to anti-mitotic drugs or to high local concentrations of IFNα (Sujobert and Trautmann, 2016; Weiss et al., 2017). To conclude on the rate of tumor growth: when tumor cells divide regularly, the tumor growth is exponential and linear on a log scale. However, this growth rate may be either accelerated, when the tumor escapes microenvironmental brakes (particularly conspicuous for SP tumors), or reduced, when angiogenesis becomes insufficient and hypoxic or necrotic regions appear.

### Tumor metabolism

Intense efforts have been devoted in the past century to answering the question of whether tumorigenesis could be a consequence of a peculiar metabolism, initially described by Otto Warburg. This important issue is mentioned only briefly here, given that no striking difference has been observed in the metabolism of TP versus SP tumors. However, it is worth underlining the mechanisms by which the metabolism of tumor cells and macrophages are linked to the vascular architecture and the spatial distribution of immune cells within the tumor.

Let us remind ourselves that the intense glycolytic metabolism in tumor cells discovered by Otto Warburg (Warburg et al., 1927) is a way for these cells to use glycolysis not only to make ATP (with a low efficacy) but also to produce building blocks necessary for the synthesis of proteins, lipids and nucleotides (Vander Heiden et al., 2009). As a by-product, they release lactate and acidify their immediate microenvironment. These phenomena contribute to organize the distribution of immune cells, in particular of the most abundant ones, macrophages. At the center of tumor islets, hypoxia, lactate and pH acidification are very high. This combination is toxic for macrophages, which prefer the more favorable microenvironment of stromal regions, close to blood vessels, where these toxic cues are minimized (Carmona-Fontaine et al., 2013; Carmona-Fontaine et al., 2017). Thus, the intratumoral localization of immune cells is strongly influenced by the global architecture of the tumor, which is itself the result of complex phenomena including gene networks dynamics, self-organization and stochastic events, as mentioned earlier.

In vitro experiments in which macrophages are co-cultured with tumor cells show that the Warburg metabolism is somehow contagious, because macrophages co-cultured in vitro with tumor cells also become more glycolytic (Penny et al., 2016). The molecular basis of this contagion is still unclear. One may hypothesize that it involves tumor-derived lactic acid, which also influences the phenotype of tumor-associated macrophages (TAM). Indeed, in the presence of tumor-derived lactate, TAM become more prone to secrete VEGF in a HIF-1α-dependent way and to express arginase 1 (Colegio et al., 2014).

Thus, the interrelated metabolism of tumor cells and macrophages contributes to the coupling between the vascular architecture and the localization of infiltrating immune cells within the tumor.

### Immune infiltrate and inflammation

As pointed out above, the *localization* of the immune tumor infiltrate is tightly linked to the vascular architecture. However, *the nature and the properties* of the immune infiltrating cells are a different issue, which will be examined now.

SP and TP tumors also differ in terms of the infiltration of TAM, usually the most abundant intratumoral immune cells. The renewal of TAMs is the result of an influx of monocytes followed by their ability to proliferate and differentiate (Franklin et al., 2014). In TP tumors, the rate of monocyte influx is very high, as the half time for intratumoral monocyte influx following adoptive transfer of fluorescent monocytes is in the order of one day (Strachan et al., 2013; Ma et al., 2014). On the basis of the supplemental data from a study performed in the MMTV-PyMT model, the monocyte influx rate can be estimated to be roughly five times slower in a SP tumor than in a TP tumor (Franklin et al., 2014). The inflammatory context of TP tumors is likely to account for the artificially accelerated influx of monocytes into these tumors. The differentiation of monocytes in TAMs may also be different in TP and SP tumors: MHCII^+^-activated TAM are more abundant in TP than in SP tumors (Guerin et al., 2019), possibly because of a larger amount of TGFβ in SP tumors, as will be discussed later.

In both TP and SP tumors, only activated TAMs may exert an anti-tumoral function, but with different efficacy in the two models. For instance, the TAM-dependent anti-tumoral action of STING agonists can only be observed in TP tumors (see below). This is also true for the anti-tumoral effect of IL-12 (Box 3).

In murine SP models, except in their inducible version, the simultaneous development of the immune system and of the tumor leads to immune tolerance (Anders et al., 2017). T cells in mice bearing SP tumors are tolerant or anergic, and thus, even after stimulation, unable to exert a strong anti-tumoral activity (Reilly et al., 2000).

Box 3. Different anti-tumoral actions of IL-12.

The anti-tumoral action of IL-12 is frequently associated with type 1 T cell responses (Weiss et al., 2007). However, IL-12 also contributes to polarize TAM towards an inflammatory, potentially anti-tumoral phenotype (Watkins et al., 2007). This may explain why the anti-tumoral effect of IL-12 is maintained against tumors grafted in *nude* mice. In particular, IL-12 secretion by intratumoral myeloid cells may exert a strong anti-angiogenic activity. Indeed, repeated intraperitoneal injections of IL-12 affects the vascular network, enough to slow down the growth of TP tumors. Again, this is true for TP but not for SP tumors (Lee et al., 2002).

Some SP tumors are characterized by the abundance of immunosuppressive *myeloid-derived suppressor cells* (MDSCs) (Sevko et al., 2013; Coffelt and de Visser, 2015), amongst which neutrophils often play an important pro-tumoral role, both in primary tumors (Di Mitri et al., 2014; Zhu et al., 2017) and in metastases (Coffelt and de Visser, 2015). Their presence may explain why an anti-tumoral effect may be obtained after adoptive transfer of antigen-specific T cells in mice bearing TP, but not SP, tumors (Zhu et al., 2017). The fact that non-stimulated neutrophils are immunosuppressive does not preclude the possibility that properly activated neutrophils may contribute to an anti-tumoral action (Di Carlo et al., 2001; Weiss et al., 2017). An immunosuppressive action may also be exerted by cancer-associated fibroblasts expressing fibroblast activation protein-α. This immunosuppression is at work both in TP (Kraman et al., 2010) and in SP tumors (Feig et al., 2013).

Inoculation with cancer cells results in massive tumor cell necrosis and in the release of tumor antigens from TP tumors that are able to trigger acute adaptive immune responses, whereas SP tumors frequently trigger chronic innate immune responses that preclude any acute T cell priming (Ciampricotti et al., 2012). It is possible to immunize a mouse against a TP tumor, but usually not against a SP tumor. This is true if the immunization is specific, that is, performed with identical tumor cells (Hirsch, 1962; Ercolini et al., 2003), with tumor-specific antigens, or following a non-specific stimulation of the immune system, for instance with Bacillus Calmette–Guérin (Old et al., 1961).

*Immunogenic cell death* (ICD) is a concept that describes the following chain of events triggered by chemotherapy. Some chemotherapeutic drugs, such as doxorubicin, not only kill tumor cells and thus release tumor antigens but also elicit the appearance of a set of danger signals including ATP, HMGB1 or calreticulin, which are released into the extracellular space or exposed at the surface of dying cells (Galluzzi et al., 2017). The combination of these events allows the triggering of a chemotherapy-induced immune response, which has been proposed to contribute to a large extent to the efficacy of doxorubicin. However, it is important to realize that the ICD concept, initially proposed by L. Zitvogel and G. Kroemer (Casares et al., 2005), has been developed in hundreds of publications on the basis of experiments that were all performed with TP tumors. The fact that it is almost impossible to elicit ICD in SP murine tumors is worrying when considering the use of this concept for human tumors (Ciampricotti et al., 2012). The immunosuppression observed in SP tumors, which is possibly a result of the abundance of TGFβ in these tumors (see below), may explain why chemotherapy on its own is not enough to elicit antitumor immune responses in SP tumors (Coffelt and de Visser, 2015). An additional weakness of the ICD concept is that, in most papers, ICD slows down tumor growth but does not induce tumor regression.

Nevertheless, the notion of ICD may be useful if it is considered in a wider sense. Indeed, if instead of relying on an ICD-based chemotherapy alone, immune cell stimulation and chemotherapy are combined, then it could be expected that the immune response may be amplified by chemotherapy-induced ICD.

### TGFβ

The last factor examined in this review, which is key to understanding differences between TP and SP tumors, is TGFβ.

The abundance of TGFβ in tumors is quite variable. TGFβ may be produced by tumor cells (Schlingensiepen et al., 2008) (and our unpublished data), by cancer-associated fibroblasts (Nazareth et al., 2007), by dying cells (Chen et al., 2001; Moustakas et al., 2002), and by TAM (Bonde et al., 2012). Of note, the secretion of TGFβ by macrophages increases after ingestion of dying cells (Fadok et al., 1998). In benign tumors or at the pretumoral state, TGFβ, together with Notch (Box 4), exerts an anti-tumoral role (Cui et al., 1994; Pu et al., 2009), which should not be forgotten.

Box 4. Anti-tumoral action of TGFβ and Notch.

The pro-quiescence function of TGFβ may be exerted together with Notch function at the interface between tumor cells and macrophages. In a study performed in a SP murine mammary tumor model (Shen et al., 2017), it has been shown that Notch may upregulate TGFβRI and thus sensitize cells to TGFβ. Conversely, TGFβ promotes the expression of JAG1, a Notch ligand, and Notch receptor activation, resulting in the production of IL-1β and CCL2 and in the recruitment of monocytes.

TGFβ is also necessary for the generation and maintenance of memory T cells (Sanjabi et al., 2009; Casey et al., 2012; Hirai et al., 2019). After an initial anti-tumoral effect of TGFβ, the dominant effect becomes protumoral through different mechanisms. One mechanism is an immunosuppression, resulting from an inhibition of activated T cells by regulatory T cells and from the anti-proliferative action of TGFβ on T cells (Fahlén et al., 2005; Esquerré et al., 2008). In addition, TGFβ activates cancer-associated fibroblasts, which contribute to neoangiogenesis, and other responses associated with wound healing (Kuperwasser et al., 2004). TGFβ is also a major inducer of EMT. Another mechanism is the acquisition by tumor cells of a resistance to TGFβ and to its anti-proliferative effects. Such a resistance may be due to an antagonism exerted by active Ras on TGFβ signaling (Fahlén et al., 2005; Burch et al., 2011).

Thus, TGFβ is a real conductor of the immunosuppressive orchestra. We have recently unraveled the existence of a new and major immunosuppressive effect of TGFβ. In the MMTV-PyMT model, despite important similarities (Varticovski et al., 2007), there is a major difference between orthotopic TP and SP tumors. Indeed, in this model, TGFβ is found in abundance and is able to block IFNβ-dependent responses in SP tumors, but not in TP tumors. In this phenomenon, TAM plays key roles, as a source and a target of both IFNβ/α and TGFβ. The TGFβ-dependent inhibition of IFNβ/α signaling may explain why SP tumors are resistant to the STING agonist DMXAA, which can trigger the regression of TP but not of SP tumors, unless mice bearing SP tumors are also treated with an anti-TGFβ antibody (Guerin et al., 2019).

The abundance of TGFβ is observed not only in classical murine SP models, but also in tumor-bearing mice in the sporadic tumor model, even though its involvement in the development of tumors in the sporadic tumor model has not been established (Willimsky and Blankenstein, 2007). In human tumors, the abundance of TGFβ has been associated with a bad prognosis (Gold, 1999; Bruna et al., 2007). TGFβ mRNA is most abundant in head and neck cancers (https://www.proteinatlas.org/ENSG00000105329-TGFB1/pathology). The next three cancers with abundant TGFβ are kidney, stomach and pancreatic cancers. For all of these cancers except pancreatic cancer, the patient prognosis is much worse for the group of patients with the highest intratumoral TGFβ level. As there is an increasing number of cancer treatments in which IFNα is at play (see e.g. Sistigu et al., 2014 for example), TGFβ-dependent inhibition of IFNβ/α signaling is likely to be involved in numerous treatment-resistance phenomena. As previously reported (Guerin et al., 2019), it would thus be of major interest to think of combining such treatments with TGFβ blockade.

**Figure 3.**
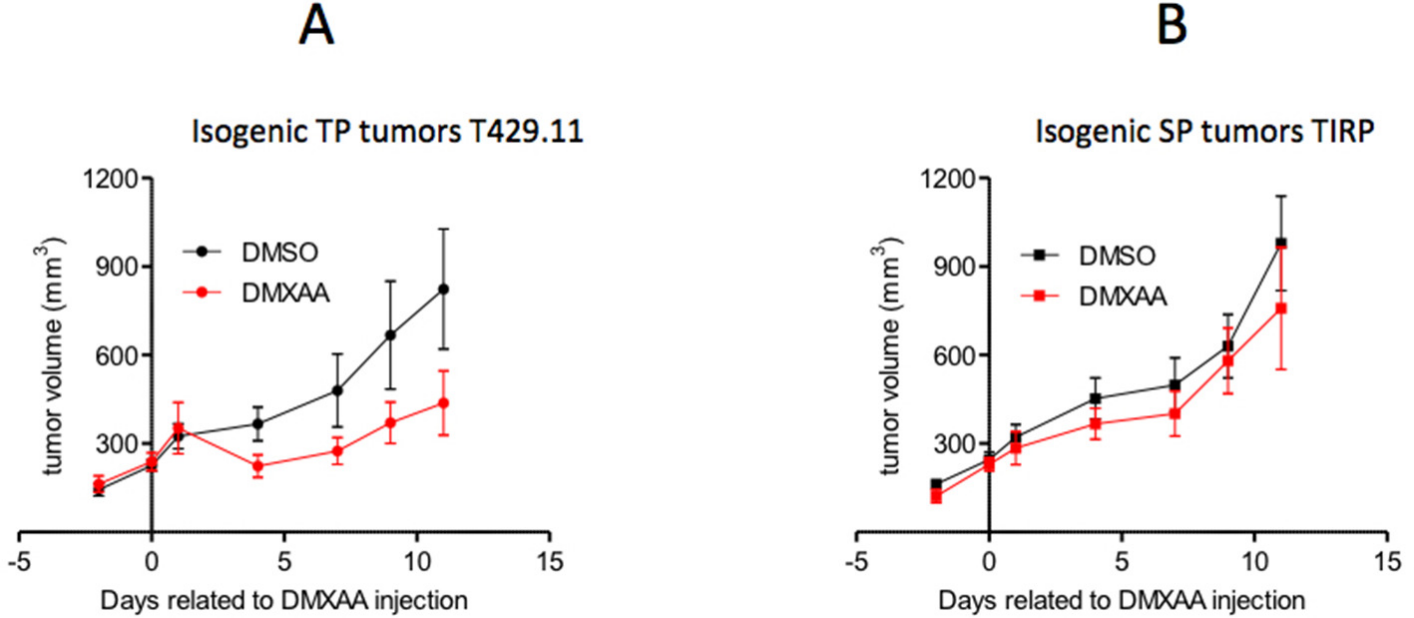
DMXAA induces a transient regression of TP melanomas but not of isogenic SP tumors. (**A**) B10.D2 mice were injected subcutaneously with 2 million T429.11 cells, a cell line derived from an induced Amela TiRP tumor (Zhu et al., 2017) (TP tumors). (**B**) TiRP mice were treated subcutaneously with 2 mg of 4-OH tamoxifen to induce tumor development as described in Zhu et al. (2017) (SP tumors). Tumor growth was measured with calipers and approximated to the volume of a spheroid (d^2^.D/2). When the average diameter was between 7 and 9 mm, T429-tumor-bearing mice or TiRP-tumor-bearing mice were randomized into two groups, with an average tumor volume of 220 mm^3^, and were treated with a single intraperitoneal (i.p.) injection of DMXAA (23 mg/kg) or DMSO (day 0). Tumor growth was monitored. Graphs show means ± S.E.M.

However, this mechanism cannot always explain the differential sensitivity of TP and SP tumors to anti-tumoral treatments. Indeed, when comparing TiRP (Huijbers et al., 2006; Zhu et al., 2017), a SP model of melanoma, and T429 tumors, its TP counterpart, one can observe that DMXAA is active against TP melanomas and not against SP melanomas (Figure 3). In this case, SP tumors also express higher levels of TGFβ than TP tumors (Fenton et al., 2001). However, contrary to what was observed in PyMT tumors, DMXAA seems to induce equal levels of IFNα signaling in both SP and TP tumors. The different sensitivity of SP and TP melanomas to DMXAA therefore relies on a different mechanism. We have mentioned above that another anti-tumoral treatment, that is the adoptive transfer of anti-tumoral-specific T cells, was active against the TP melanoma but not the SP melanoma. This differential sensitivity appears to be due to the larger abundance of MDSCs in the SP melanoma (Zhu et al., 2017). These MDSC mediate immunosuppression by triggering the apoptosis of tumor-infiltrating CD8 T cells through the Fas/Fas-ligand pathway (Zhu et al., 2017). Whether or not the differential response to DMXAA is also MDSC-dependent remains to be clarified.

A third example of identical tumor cells that behave differently in a SP and a TP context are represented by the *Kras^G12D/+^*;*Trp53^R172H/+^*;*Pdx-1-Cre* (KPC) mice, which develop spontaneous pancreatic ductal adenocarcinoma (PDAC), and the KPC-derived PDAC cell line. There too, treatment of mice with a chemotherapy plus an anti-CD40 antibody results in a conspicuous T cell infiltrate in TP but not in SP KPC tumors (Beatty et al., 2015). The reason for this difference has not been elucidated.

## Organ-specificity of tumors

It is often considered that different organs give rise to different types of human cancers that have distinct genomic profiles and sensitivities to anticancer therapies. The origin of these differences has been explored with different approaches including: (i) profiling the tumor-associated mutations and the transcriptional landscape of the immune infiltrate; and (ii) considering the original functioning of the healthy organ and the cross-talk between tumor cells and the stromal and immune microenvironment. Here, we examine factors that could be decisive for the organ-specificity of human tumors, and consider which preclinical tumor models should be used to take this specificity into account.

### High-throughput analyses

To what extent can sets of mutations in tumors be viewed as organ-specific? A few mutations are found more frequently in some organs than in others. For instance, ∼50% of melanomas harbor recurrent BRAF mutations, which are key to the immune evasion of melanoma (Sumimoto et al., 2006) and which are amenable to specific treatments (Robert et al., 2015), whereas such mutations are much less frequent in colon cancers (Corcoran et al., 2012). By contrast, colorectal cancers frequently harbor KRAS mutations, which are rare in melanomas (AACR Project GENIE Consortium, 2017) but extremely frequent in pancreatic cancers (Lanfredini et al., 2019). A thorough analysis of the mutanome of >60,000 tumors has demonstrated, however, that *tumors have remarkably few shared alterations with other tumors, even in the context of major driver alterations and in specific disease types* (Hartmaier et al., 2017). Thus, mutanome profiles are too variable and overlapping to constitute major determinants of the organ-specificity of tumors.

What about the global mutational burden? It is usually considered that a higher mutational load means that a tumor will produce a larger number of neoantigens, leading to a higher immunogenicity of the tumor. The average mutational burden, or number of neoantigens, is approximately 10 times higher in melanoma or lung squamous carcinoma than in bone cancer, germ cell tumors or thyroid cancer (Alexandrov et al., 2013; Schumacher and Schreiber, 2015; AACR Project GENIE Consortium, 2017). Overall, highly mutated tumors do indeed have a better respone rate to immunotherapeutics (Hugo et al., 2016). However, there is also a substantial number of patients who have a good immune response despite a low mutational load, and vice versa (Wellenstein and de Visser, 2018). What appears to be decisive for the efficacy of checkpoint inhibitors is the pre-existing immune context, including T cell infiltrate and the presence of immune-costimulatory molecules (Tumeh et al., 2014; Auslander et al., 2018), which is often but not necessarily related to the mutational burden.

Are there immune profiles that are specific to tumors in a given organ? This appears as an interesting hypothesis, since both the anatomic location and the functions of an organ constrain the type of pathogen that may invade them, and therefore how the immune system has evolved to adapt host defense accordingly. In particular, the abundance of the different types of resident immune cells is quite variable from organ to organ in healthy tissues. However, the variability in the abundance of immune cells observed amongst, say, lung tumors is much larger than that observed in the immune profile of healthy lungs (Pao et al., 2018). Because of this tumor-to-tumor variability for a given organ, and even though there is a definite influence of an organ on its immune infiltrate in healthy conditions, one cannot say that one tumor immune profile is organ-specific.

Thus, these approaches have provided a huge amount of data on many tumor types. However, *owing to the descriptive nature of these analyses, these studies do not yield mechanistic insights* and they have not led to strong predictive conclusions on tumor-specific treatments (Wellenstein and de Visser, 2018).

### Functional points of view

One may wonder if there is a functional explanation behind the fact that the average mutational burden is much higher in melanoma or lung squamous carcinoma than in thyroid or bone cancer. We propose here such an explanation. It is now clear that several key mutations are necessary but not sufficient for a tumor to develop. Tumor development also requires a favorable, tumor-promoting microenvironment, as thoughtfully predicted in the ‘*seed and soil*’ theory of S. Paget in 1889, a theory often revisited since then (Fidler, 2003). A poly-mutated transformed cell is initially prevented from proliferating by a protective microenvironment that includes not only the immune system but also the normal stroma (Mueller and Fusenig, 2004; Bissell and Hines, 2011). One may hypothesize that tumors with the highest mutation load are those on which the initial anti-tumoral brake was the strongest, so that a large number of mutations were needed for the nascent tumor to escape from its anti-tumoral microenvironment. Thus, melanoma or lung carcinoma would appear in a context that is quite unfavorable for tumor development, whereas, at the other end of the mutational burden spectrum, bone cancer or germ cell tumors would emerge in a less constrained environment (AACR Project GENIE Consortium, 2017). This could be related to the fact that both the skin and mucosa represent interfaces between the inside and outside of the organism, where both the exposure to pathogens or other insults and the immune surveillance against them are greatest.

In such a view, a mutational burden would be a measure of the efficacy of local immune surveillance rather than a decisive tumor cause in itself. The key determinant here would be the anatomical location of the organ and its associated functions and exposure to insults.

In the whole review, we have underlined the importance of structure–function relationships in the tumor microenvironment. It appears that this issue is probably also a key factor in the organ specificity of tumors. This is true for all the dimensions that we have taken into account: general architecture of the tumor, structure of the vascular tree, and stroma development, all of these elements influencing in turn the immune infiltrate. It is in this structure that a cellular network including tumor, stromal, immune and endothelial cells self-organizes, with the growth factors and inhibitors produced by some cells influencing the development of other ones. This network is likely to be organ-specific, because the dominant resident cells and growing factors are not the same in all organs.

To illustrate the relative importance of mutations or gene expression on one hand, and anatomical location and function on the other, one can observe that the frequency of mutated KRAS is high in colon cancer, and very high in pancreatic cancer. However, the very different prognosis for the two tumors underlines that a mutated KRAS cannot explain the very bad prognosis for pancreatic tumors. What is striking and specific in pancreatic tumors is an exuberant stroma, including specific fibroblasts (pancreatic stellate cells), amorphous fibers, plus a macrophage infiltrate, and an intense fibro-inflammatory response that generates a very high interstitial fluid pressure (Zhang et al., 2018). In addition, pancreatic tumors show a particularly defective vascularization (Provenzano et al., 2012), leading to hypoxia and its consequences. One may hypothesize that it is the combination of mutated KRAS with these organ-specific features (including organ-specific growth factors) that results in a particularly nasty cancer.

Which conclusions could then be drawn concerning optimal preclinical tumor models ? First, we have to recognize that we are unable, in murine models, to address properly an important issue: human tumors usually take years to start really growing, and this very long latency phase, during which mutations probably accumulate, is impossible to recapitulate in murine tumors that could be used conveniently in large numbers. Second, there is an organ specificity for tumors that is associated with the specificities of organs: anatomical location, functions, dominant cellular and molecular actors. Thus, when working on preclinical models for potential breast cancer treatments, it makes sense to use either spontaneous mammary tumors or dissociated mammary tumors transplanted orthotopically. By contrast, with the perspective of treating pancreatic cancer, a tumor cell line of pancreas or lung origin, implanted subcutaneously, will be of low relevance, especially if it is a post-EMT cell line that prevents the formation of a typical tumor structure with tumor islets and a conspicuous stroma. Nevertheless, many results, and some of them quite interesting, have been obtained with such imperfect approaches.

## Conclusions

All animal models have advantages and drawbacks, none of them may be considered as ideal. However, authors have a marked propensity to mention the advantages of the model they use, and too often choose not to discuss its drawbacks. In this review, the strengths and weaknesses that are inherent to specific tumor models are analyzed.

Sporadic tumor models are certainly the closest to human tumors, but their slowness to develop and the large variability in their growth make them barely usable, in practice, for systematic studies.

SP mammary tumors arising in female C3H mice almost fall into the same category, but are somehow more simple to use, especially if the probability of tumor occurrence is increased by infecting the mice with MMTV (Fenton et al., 2005).

GEMM, characterized by the expression of several oncogenes in a specific tissue, represent an interesting compromise. They keep proximity to human tumors, even though their growth rate is faster. They present important constraints of use, albeit less huge than for sporadic models.

TP tumors constitute the most popular tumor model. As pointed out throughout this review, they differ profoundly from tumors that grow spontaneously in humans or in SP murine models. The difference with SP tumors is larger for tumors growing after the injection of a cell line than for those formed after the injection of the various cells dissociated from a spontaneous tumor. The difference with SP tumors is also larger for tumors that are implanted subcutaneously rather than orthotopically. Another difference may be observed between tumors implanted subcutaneously or intradermally (Joncker et al., 2016). The main ways in which TP and SP tumors differ are summarized below and in Table 1.

The vascularization of TP tumors is not dependent on an angiogenic switch, which has already taken place in implanted cells. Thereafter, the growth rate of the vascular network is usually faster in TP tumors than in SP tumors, contributing to the frequently faster growth rate of TP tumors. However, the rapid growth of the vascular tree of TP tumors makes it more immature and more fragile than that of SP tumors.

In TP tumors, potential anti-tumoral T cells differentiate without the tolerization phase that takes place in SP murine tumors and in human tumors. The myeloid infiltrate is more immunosuppressive in SP tumors for several reasons. One may be a larger abundance of neutrophil attractants, for reasons which remain to be determined (Zhu et al., 2017). Another reason may be a larger concentration in some SP tumors (Guerin et al., 2019; Zhu et al., 2017) of TGFβ, which polarizes TAM towards a more immunosuppressive state.

These strong conclusions could be drawn because we have been able to compare, for two tumor types (mammary and melanoma), isogenic tumor cells in the context of both TP and SP tumors. The conclusions obtained from the two systems were not identical but were complementary. It would be of major interest to extend such a comparison to additional pairs of SP and TP tumors.

All of these features may contribute to explain the different sensitivity of TP and SP murine tumors to a large spectrum of anti-tumoral treatments, which work better (and sometimes only) in TP tumors (Table 1). This is the case for the anti-tumoral effect of cortisone (Burton and Begg, 1961), the efficiency of the adoptive cell transfer of antigen-specific T cells (Zhu et al., 2017), practically all the ICD-based chemotherapies, and the anti-angiogenic effects of IL-12 (Lee et al., 2002) or DMXAA.

Erroneous conclusions may be drawn from studies with TP tumors. For instance, tumors growing after implantation of B16 melanoma lines, or of LLC or MAD109 lung tumor cell lines, are poorly immunogenic and resistant to immunotherapy, in contrast to the responses observed in the corresponding tumors in patients (Zeiser et al., 2012). These observations do not imply that transplanted murine tumor models should no longer be used, but they indicate that data translation from these models to human studies without an intermediate validation in SP models may be risky. Although this seems to be an elementary caution, it has not always been taken, for instance, for the phase III clinal trial with DMXAA (Lara et al., 2011). We have also pointed out that, in many cases, human xenografts appear to be a false good idea, because they represent a situation in which the multiple interactions between tumor cells, T cells, macrophages, stromal cells and endothelial cells cannot be properly reconstituted. Finally, in order to be able to use certain molecular tools, research has been more and more concentrated on a few strains of laboratory mice. It is reasonable to think that diversifying the animal models used in research would help to propose more efficient anti-tumoral treatments.
